# An Interesting Cause of Hyperandrogenemic Hirsutism

**DOI:** 10.1155/2014/987272

**Published:** 2014-12-16

**Authors:** Murat Atmaca, İsmet Seven, Rıfkı Üçler, Murat Alay, Veysi Barut, Yaren Dirik, Yasin Sezgin

**Affiliations:** ^1^Department of Endocrinology and Metabolism, Yuzuncu Yil University Faculty of Medicine, 65100 Van, Turkey; ^2^Department of Internal Medicine, Yuzuncu Yil University Faculty of Medicine, 65100 Van, Turkey

## Abstract

Mild clinical signs of hyperandrogenism such as hirsutism may appear during the menopausal transition as part of the normal aging process, but the development of frank virilization suggests a specific source of androgen excess. We report a case of a 68-year-old woman with signs of virilization that had started 6 months before. Clinical analyses revealed high levels of serum testosterone for a postmenopausal woman. Pelvic MRI and abdomen CT showed no evidence of ovarian and adrenal tumor. Postmenopausal hyperandrogenism can be the result of numerous etiologies ranging from normal physiologic changes to ovarian or rarely adrenal tumors. Our patient was found to have iatrogenic hyperandrogenism. This condition is rarely reported cause of virilization.

## 1. Introduction

The postmenopausal ovary remains hormonally active and secreting significant amounts of androgens and relatively less estrogens many years after menopause [1]. Although estrogen levels are reduced abruptly after menopause, androgen secretion declines gradually with aging and is maintained until later stages of life [2, 3]. Androgen secretion in pre- and postmenopausal ovaries depends on LH stimulation. The very high gonadotropin levels of menopause could maintain ovarian androgen production [2, 3]. This imbalance between estrogens and androgens after menopause is amplified further by the evolving decrease in sex hormone binding globulin (SHBG) concentrations and the subsequent increase of free androgen index [4]. As a result, menopause may be accompanied by the appearance of a few terminal hairs in the face and by a decrease in body and scalp hair that can be considered part of the normal menopausal process [5].

The development of true hirsutism (defined as the presence of excessive terminal hair in androgen-dependent areas), alopecia, or acne should not be considered normal in postmenopausal women. The main differential diagnosis of androgen excess in postmenopausal women is either tumoral causes, namely, androgen-secreting ovarian or adrenal tumors, or nontumoral causes, that is, polycystic ovarian syndrome, hyperthecosis, Cushing's syndrome, congenital adrenal hyperplasia, and iatrogenesis [6].

When hirsutism is accompanied by signs of virilization such as severe balding, deepening of the voice, or clitoromegaly, an underlying androgen-secreting tumor that may be malignant must be ruled out.

Here, a 68-year-old postmenopausal case with virilization is presented with a discussion referenced to the medical literature.

## 2. Case Report

A 68-year-old female was admitted in our clinic with complaints like gradually increased hirsutism and deepening of the voice. She was menopause at 52 years old and she has eight children. The medical history of the case showed that she was followed up due to hypertension for 5 years and had good tension control with telmisartan 80 mg daily. Also medical history of the case showed that she was diagnosed with osteoarthrosis and used some creams which contain probably nonsteroidal anti-inflammatory drugs but she does not remember the names of this drugs. She did not have any outstanding condition in family history. Her arterial blood pressure was measured as 140/90 mmHg, pulse was 84 beats/min, and body temperature was 36.9°C in her physical examination. Her face was plethoric and in her skin there was hirsutism especially in upper lip, chin, and upper abdomen areas. In these areas Ferriman-Gallwey score was 4. Abdomen and cardiovascular and respiratory system were also observed to be normal. There was no clitoromegaly or temporal baldness. In the hemogram of our case, polycythemia was observed (hemoglobin: 18 g/dL, hematocrit 54%). The electrolyte levels and biochemical levels were revealed to be normal. Testosterone was found to be 25,40 nmol/L in our case who was suspected to have hyperandrogenism due to the existent clinical pattern (testosterone normal range for women: 0,38–1,97 nmol/L). The other hormonal parameters were shown in Table 1. The case was hospitalized for differential diagnoses of hyperandrogenism including polycystic ovary syndrome (PCOS), congenital adrenal hyperplasia (CAH), Cushing's syndrome, and benign and malignant androgen-secreting ovarian and adrenal tumors. 17 OH progesterone level was detected 302,57 pmol/L and diagnosis of CAH was excluded. 1 mg dexamethasone suppression test was detected 2,7 nmol/L and Cushing's syndrome was excluded. Pelvic MRI and abdomen CT for androgen-secreting ovarian and adrenal tumors revealed that there was no adrenal and ovarian mass. In this imaging study right and left ovaries sizes, respectively, were 22 × 12 mm and 22 × 14 mm and adrenal configuration and size were normal. On the 4th day of her hospitalization, the control testosterone levels were detected 12 nmol/L. Testosterone levels were decreased every other day, respectively, 7,4 nmol/L, 4,3 nmol/L, and 2,1 nmol/L. This gradual decrease in testosterone levels was suggested to be due to exogenous androgen exposed. In our interview with the patient's husband it was showed that he has used transdermal testosterone gel since a year for impotence. Hyperandrogenism in our cases could be passed from her husband application region by skin contact. Our patient's husband has hidden his illness from patient and he said that he uses this gel for myalgia. Our patient has been using the husband's transdermal testosterone gel unconsciously for knee pain for 6 months.

## 3. Discussion

Mild hirsutism and alopecia in postmenopausal women can be considered part of the normal menopausal process [5]. The development of true hirsutism, alopecia, or acne should not be considered normal in postmenopausal women. The main differential diagnoses of androgen excess in postmenopausal women are polycystic ovary syndrome (PCOS), congenital adrenal hyperplasia (CAH), Cushing's syndrome, benign and malignant androgen-secreting ovarian and adrenal tumors, and iatrogenic causes [6].

PCOS is the most common cause of hyperandrogenism in women at reproductive age and diagnostic criteria are well defined in premenopausal women [7]. However, PCOS in postmenopausal women is not well defined, as no robust criteria are currently available and only recently age-based criteria for the definition of PCOS have been proposed [7–9]. In women with PCOS symptoms usually start in adolescence and gradually progress during reproductive years [10]. Our case's hirsutism occurred six months ago and deepening of the voice is concomitant with this symptom. Virilization symptoms and very high testosterone levels suggested excluding PCOS.

Congenital adrenal hyperplasia (CAH) is another cause of hyperandrogenism. Enzymatic deficiencies causing adrenal hyperplasia and excessive androgen production are mainly due to 21-hydroxylase and rarely to 3*β*-hydroxysteroid dehydrogenase and 11*β*-hydroxylase deficiencies [11–13]. Most of CAH cases are diagnosed and treated at a much younger age [14]. High testosterone levels such as our cases can be seen in simple virilizing or salt losing forms of CAH; however, these forms of CAH are associated with adrenal insufficiency and high ACTH, both of which the patient did not have.

Nonclassic adrenal hyperplasia (NCAH) arising from 21-hydroxylase deficiency is found in 1–10% of hyperandrogenic women depending on ethnicity and is associated with elevated 17-hydroxy-progesterone and adrenal androgen levels [11]. The prevalence of hirsutism in women with NCAH increases with age and the degree of hyperandrogenism may worsen in postmenopausal women [15]. In our case's medical history hirsutism occurred six months ago and before six months there was not any compliant of hirsutism. In reproductive her menscycles are regular along reproductive age. There was no adrenal hyperplasia or nodular lesion in abdomen CT and 17 OH progesterone levels were normal range. For these reasons NCAH was excluded.

Cushing's syndrome (CS) may also be diagnosed after menopause and cause symptoms or signs of androgen excess. Hirsutism can be found in approximately 50% of patients with CS mainly attributed to adrenal androgen excess; endogenous hypercortisolism also correlates positively with free androgen levels probably due to SHBG reduction [16]. In contrast to CS secondary to adrenal carcinomas, signs of hyperandrogenism are usually mild in women with the adrenocorticotropin- (ACTH-) dependent CS and are virtually absent in women with adrenal adenomas [17]. There was no adrenal mass in adrenal CT and 1 mg dexamethasone suppression test was detected 2,7 nmol/L and Cushing's syndrome was excluded.

When hirsutism is more severe and accompanied by symptoms or signs of virilization, ovarian hyperthecosis, adrenals, or the ovarian tumors should be excluded [6, 18].

The main nontumoral cause of postmenopausal ovarian biochemical hyperandrogenism seems to be ovarian hyperthecosis [19]. Hyperthecosis is a severe form of PCOS and results from an overproduction of androgens in the ovarian stromal cells [20]. Women typically present with slowly progressive acne and hirsutism (e.g., excessive male pattern hair growth), and they are likely to be virilized [21]. Although the exact aetiology is unclear, some authors claım that hyperthecosis ın postmenopausel women originate from elevated gonodotrophin productions. Patients with hyperthecosis typically have normal serum dehydroepiandrosterone sulfate (DHEA sulfate) concentrations [22, 23]. Ultrasonography in women with hyperthecosis usually shows a bilateral increase in ovarian stroma and the ovaries appear more solid [24]. Similarly MRI findings in ovarian hyperthecosis include symmetric bilateral ovarian enlargement [25]. Serious hyperandrogenemia, virilization symptoms, and normal DHEAS levels suggested hyperthecosis in our case but in pelvic MRI showed atrophic ovaries.

Tumorous hyperandrogenism is suspected when testosterone levels are greater than 3,5–5 nmol/L and is usually associated with abrupt onset and rapid evolution of symptoms or virilization [26]. Adrenal androgen-secreting tumors are rare but highly suggestive of malignancy. These tumors are easily identified on imaging techniques. Patients with adrenal androgen-secreting tumors typically have elevated serum dehydroepiandrosterone sulfate (DHEA sulfate) concentrations [27]. In our case DHEAS levels was normal and adrenal imaging did not detect any mass.

Androgen-producing ovarian tumors include lipoid, Leydig cell, granulosa-theca cell, and Sertoli-stromal cell tumors. These tumours are rare representing only 10% of all ovarian tumours [28, 29]. Ovarian androgen-secreting tumors are not easily identified in imaging techniques. Their ultrasonographic aspect depends on tumor type and ultrasonography studies for Sertoli-Leydig, steroid cell tumors, and thecomas have been reported negative in postmenopausal women [30–33]. Rapidly progressive virilization symptoms and very high testosterone levels suggested tumoral causes. Negativity of ovarian imaging does not exclude ovarian malignancy. Ovarian sampling, bilateral oophorectomy, or GnRH analogue treatment is suggested in these cases. Ovarian sampling is very difficult and invasive and, even at the best centers, sampling can accurately localize the ovarian tumor only 66% of the time [34].

We detected serious decrease in control testosterone levels. This condition, before further investigation, was suggested exogenous androgen exposed. Testosterone levels were decreased gradually every other day. Our patient has been using the husband's transdermal testosterone gel unconsciously for knee pain for 6 months. Iatrogenic or self-administration of androgenic drugs and supplements can induce symptoms and signs of hyperandrogenism via an increase in circulating androgen levels or an intrinsic androgenic activity of the drug. Drugs most commonly responsible include androgens, anabolic steroids, and antiepileptics [35]. Several case reports show that topical androgens may result in clinical syndromes of hyperandrogenism in exposed children and women [36].

In conclusion, iatrogenic causes of hyperandrogenemia should be kept in mind in differential diagnosis of hyperandrogenemia. Hospitalization to prevent from exposure and sequential testosterone level measurement can help in this condition's diagnosis.

## Figures and Tables

**Table 1:** Hormonal profiles of case.

	Normal range	Result
Follicle stimulating hormone	26,7–133,41 IU/L	30,09 IU/L
Luteinizing hormone	10,39–64,57 IU/L	13,04 IU/L
Estradiol	0–73,42 pmol/L	55,06 pmol/L
Total testosterone	0,38–1,97 nmol/L	25,40 nmol/L
Sex hormone binding globulin	19,8–155,2 nmol/L	43,1 nmol/L
Progesterone	0–636 pmol/L	318 pmol/L
17 OH progesterone	605–2723 pmol/L	302,57 pmol/L
Adrenocorticotropin hormone	0–10 pmol/L	3,3 pmol/L
Cortisol	100–535 nmol/L	330 nmol/L
1 mg dexamethasone suppression test	0–50 nmol/L	2,7 nmol/L
Dehydroepiandrosterone sulfate	270–540 nmol/L	508 nmol/L
